# Reduced reward responsiveness in treatment resistant depression of middle-aged adults: Association with carotid artery stiffness and tetrahydrobiopterin

**DOI:** 10.1371/journal.pone.0290784

**Published:** 2023-08-31

**Authors:** Kalpana K. Barhwal, Barsha Parida, Jigyansa Pattnaik, Praveen Rowlo, Sudipta Mahakud, Suravi Patra, Bodepudi N. Rao, Bidhubhusan Mahapatra

**Affiliations:** 1 Department of Physiology, All India Institute of Medical Sciences (AIIMS), Bhubaneswar, India; 2 Department of Physiology, Kalinga Institute of Medical Sciences (KIMS), Bhubaneswar, India; 3 Department of Psychiatry, Kalinga Institute of Medical Sciences (KIMS), Bhubaneswar, India; 4 Department of Biomedical Sciences in Dentistry, Hebrew University of Jerusalem, Jerusalem, Israel; 5 Department of Radiology, All India Institute of Medical Sciences (AIIMS), Bhubaneswar, India; 6 Department of Psychiatry, All India Institute of Medical Sciences (AIIMS), Bhubaneswar, India; 7 Norwegian Refugee Council, Amman, Jordan; University of Pittsburgh School of Medicine, UNITED STATES

## Abstract

Nearly one third of the population diagnosed with major depressive disorder (MDD) fail to respond to two or more antidepressant drugs of adequate dose and duration. This necessitates identification of confounding psychological and physiological factors that could contribute to treatment resistant depression (TRD). The present longitudinal study investigated the influence of behavioural inhibition system (BIS) and behavioural approach system (BAS) in treatment resistance. Further, the association of depression severity with physiological factors contributing to arterial stiffness was also investigated. Baseline data was acquired from 101 middle-aged (36–56 years) patients on immediate diagnosis with MDD using DSM-V criteria. Follow ups were conducted at 06 months and 12 months during treatment. Psychological assessment battery at baseline and follow ups comprised of Hamilton depression rating (HAM-D) for depression severity, WHODAS-2 and BIS-BAS score. Atherosclerosis and central arterial stiffness were measured by intima-media thickness of internal carotid artery and brachial-ankle pulse wave velocity. Physiological factors influencing central vascular function viz., body-mass index, estimated glomerular filtration rate, HbA1c, central systolic and diastolic blood pressure, heart rate and tetrahydrobiopterin were also investigated. Our results show lower reward responsiveness (BAS-RR) and higher BIS scores in TRD patients along with differentially higher intima-media thickness of left internal carotid artery. Higher depression severity at all stages of the study was correlated with lower tetrahydrobiopterin and BAS-RR scores. We, therefore, suggest that vascular depression resulting due to increased intima-media thickness of left carotid artery and lower tetrahydrobiopterin could be contributing factors for treatment resistance in middle-aged MDD patients.

## 1. Introduction

Major Depressive Disorder (MDD) is the leading contributor to global burden of psychiatric disorders. It is characterized by persistence of negative thoughts and emotions leading to decreased psychosocial functions and behavioural abnormalities [1]. Nearly 45% of MDD patients do not respond appropriately to at least two mechanistically dissimilar antidepressant treatments and are categorized as treatment resistant [2]. This high prevalence of treatment resistance can be attributed to the heterogeneous state, multifaceted causal mechanisms and hitherto unidentified patho-physiological changes leading to MDD. Hence, considerable effort is being made worldwide for exploring physiological and biochemical factors to identify novel therapeutic targets and strategies for treatment resistant depression (TRD).

The positive or negative mood state is conceptualized to be regulated by two diametrical motivational systems in the brain that regulate sensitivity to reward (Behavioural Activation System-BAS) or punishment (Behavioural Inhibition System-BIS) [3]. Recent studies using psychological assessment tools and brain imaging have established pivotal role of motivational systems in depression and affective disorders. Depressed patients show higher BIS and lower BAS-reward responsiveness which is related to severity of depressive traits [4–6]. The physiological basis of reduced reward responsiveness in depression and the implications of higher BIS or lower BAS scores on treatment resistance in MDD patients, however, still remains obscure.

Arterial stiffness and vascular risk factors resulting in vascular depression and altered cerebral perfusion are gaining importance as physiological contributing factors for MDD in elderly population [7]. Growing evidence from magnetic resonance image (MRI) based studies demonstrate the bidirectional relationship between cerebrovascular risk factors and depression [8]. Vascular depression has lately gained acceptance as a subtype of late-life depression in elderly population (> 60 years), characterized by a distinct clinical presentation and its association with cerebrovascular damage. Studies by Gonzalez et al (2012) show association of vascular depression with higher degree of functional impairment when compared to non-depressed population and adults meeting criteria for major depression alone [9]. Dregan et al (2020), during UK biobank population study, reported significant direct association between depression and arterial stiffness index levels in middle aged population [10]. Similar observations on association of depressive symptoms with arterial stiffness in subjects with blood pressure and comorbid diabetes mellitus were made by Peng et al (2020) in Chinese population [11]. The association of vascular factors with severity of depression and treatment resistance in middle-aged Indian population, however, remains less studied. Treatment of MDD resulting due to arterial stiffness and vascular factors, on the other hand, may require alternate approaches aimed at improving cerebral circulation [12,13]. Hence, there is a need to investigate the association of treatment resistance in middle-aged MDD patients with central arterial stiffness and related vascular factors.

Several studies have reported on the role of body mass index (BMI), glomerular filtration rate, circulatory glucose and tetrahydrobiopterin in arterial stiffness [13,14]. Tetrahydrobiopterin, in particular, influences vascular elasticity through activation of endothelial nitric oxide synthase (eNOS) and could have potential diagnostic significance for vascular depression in TRD [15]. Non-invasive brachial-ankle pulse wave velocity and intima-media thickness of carotid arteries are known measures of central arterial stiffness and early atherosclerosis [16]. Previous studies measuring carotid to femoral pulse wave velocity (cfPWV) showed the association of aortic stiffness with MDD and depressive symptoms among middle-aged men [17]. However, association of central arterial stiffness with treatment resistance in middle-aged MDD patients and its influence on psychological measures related to behavioural motivational systems remains to be established.

The present study was conducted to bridge the hiatus in knowledge on association of behavioural motivational systems and vascular factors with treatment resistance and severity of depression in middle-aged MDD patients. A longitudinal study was designed to identify the magnitude of difference between BIS-BAS scores and vascular factors in responders and non-responders at baseline and during follow-ups at 6 months and 12 months during the course of treatment. Association of depression severity with central arterial stiffness and its confounding physiological and metabolic factors was also determined to identify differential contribution of these factors to treatment resistance.

## 2. Methods

### 2.1 Participants

The study was performed in accordance with the principles stated in the Declaration Criteria of Helsinki and with approval of the Institutional Ethical Committee(IEC) (IEC No. T/IM-F/18-19/02). Written informed consent was obtained from each patient prior to the study.

**Fig 1:**
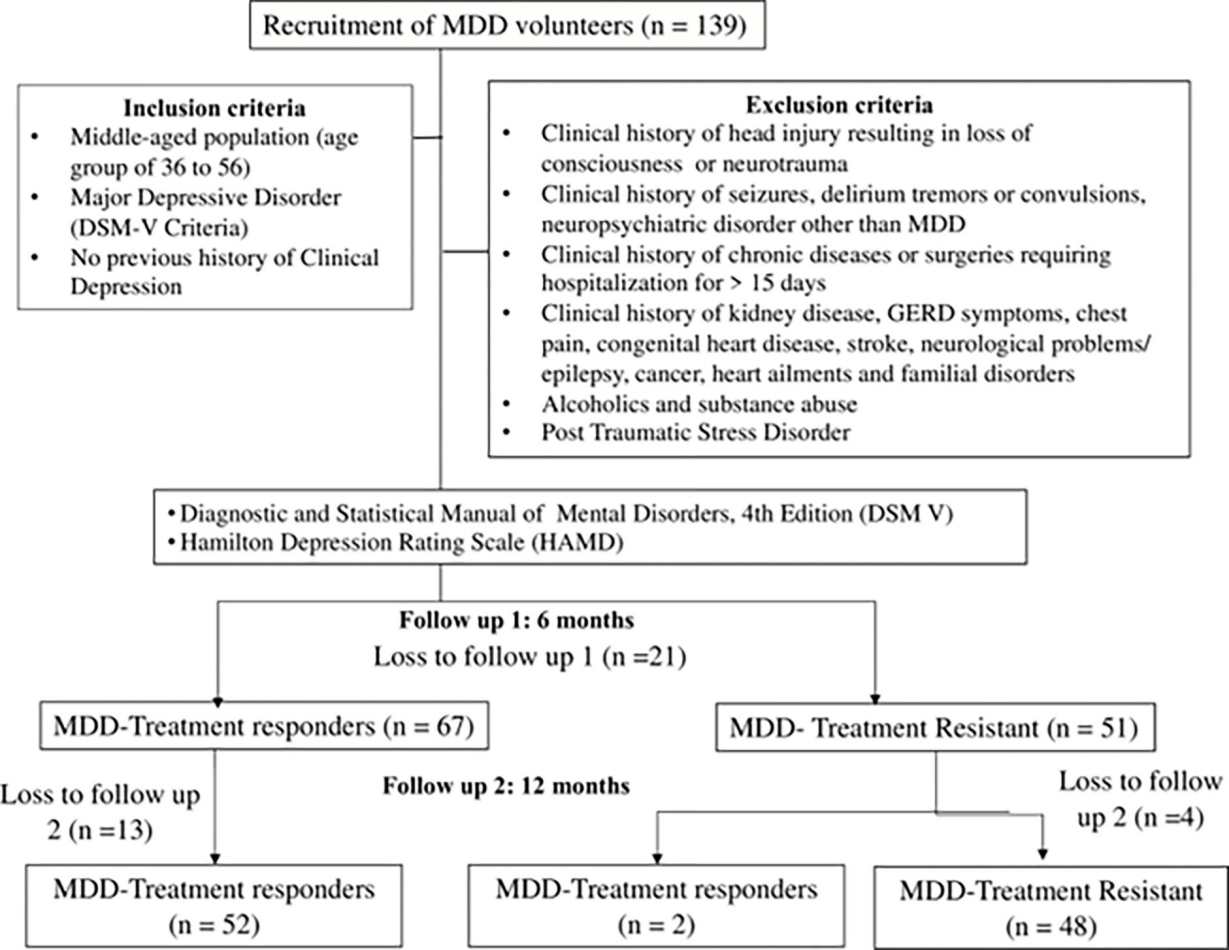
Study design and timelines for follow ups.

139 middle-aged patients (36 to 56 years) with depressive symptoms visiting Department of Psychiatry, AIIMS, Bhubaneswar were enrolled for the study. Clinical diagnosis of MDD was done based on Diagnostic and Statistical Manual of Mental Disorders, 5^th^ Edition (DSM-V) and Hamilton Depression Rating Scale (HAM-D) was applied for determining severity of depressive symptoms [18]. During the first and second follow-up at intervals of 06 months each, MDD patients were categorized to MDD responders and MDD treatment resistant based on classification criteria for treatment resistant depression (TRD) viz., Stage 0 i.e. MDD responders and Stage 1–4 i.e. MDD treatment resistant [19] (Fig 1).

### 2.2 Study design

A prospective longitudinal study was conducted on Stage 1 and Stage 2 TRD patients. The study design involved recruitment of newly diagnosed MDD patients as volunteers and acquisition of baseline data psychological and physiological data prior to anti-depressant treatment. Details of the patient recruitment process are presented in Fig 1.

After acquisition of baseline data, patients then followed the standard treatment regime for depression that included clinician prescribed selective serotonin reuptake inhibitors (SSRIs). Those not responding to SSRIs during regular clinical follow ups were shifted to serotonin and norepinephrine reuptake inhibitors (SNRIs). Follow-up studies were conducted at intervals of 6 months and 12 months during treatment. MDD subjects were categorized into responders and treatment resistant during the follow ups based on the remission of depressive symptoms as determined by >50% decrease in HAM-D scores when compared to baseline or previous follow-up HAM-D scores. Socioeconomic status was assessed using modified Kuppuswamy Scale [20], and health and disability was assessed using world health organization (WHO) disability assessment schedule 2.0 [21]. Baseline and follow up data of patients who participated throughout the study was considered for statistical analysis.

### 2.3 Psychological measures

#### 2.3.1 Diagnostic and statistical manual of mental disorders(DSM)-V

Diagnosis of MDD was performed by clinical psychiatrist using the structured clinical interview for DSM-V [22]. Major depressive episode was diagnosed when a person had persistent depression or hopelessness or anhedonia for more than 2 weeks along with any five of the nine additional criteria of depression [23] viz. (1) depressed mood (subjective or observed) (2) loss of interest or pleasure, most of the day (3) change in weight or appetite (4) insomnia or hypersomnia (5) psychomotor retardation or agitation (6) loss of energy, or fatigue (7) worthlessness or guilt (8) impaired concentration or indecisiveness (9) recurrent thoughts of death or suicidal ideation or attempt.

#### 2.3.2 Hamilton Depression Rating Scale (HAM-D)

Severity of depression was assessed using the HAM-D depression rating scale which provides a clinician-rated measure for severity of depression, changes in severity of depression over time and efficacy of treatment [18]. HAM-D as an inventory for MDD has been reported to have satisfactory reliability with an alpha coefficient of 0.81 and intra-class correlation coefficient for inter-rated reliability of 0.95 [24]. HAM-D scores < 7 was considered normal while scores between 8–13 were categorized as mild depression, 14–18 as moderate depression, 19–22 as severe depression and scores > 23 indicated very severe depression.

#### 2.3.3 Behavioural Inhibition and Activation Scales (BIS/BAS)

BIS/BAS scales were applied to assess the behavioural responses of depressed and non-depressed participants towards approach or reward system (BAS) and withdrawal or punishment system (BIS) [25,26]. The BIS/BAS scale is a widely used, 20-item questionnaire with good reliability and convergent and discriminant validity [27–29]. It assesses the reaction of individuals to certain situations and has four subscales viz., Behavioral Inhibition (BIS); Behavioral Activation Reward Responsiveness (BAS–RR), Drive (BAS–Drive), and Fun-Seeking (BAS–Fun). The BIS generally regulates aversive motivation, and is therefore, sensitive to signals of punishment and non-reward related to experience of negative feelings such as fear, anxiety, frustration and sadness [30]. On the contrary, BAS regulates appetitive motivation and is sensitive to signals of reward, non-punishment and is therefore responsible for experience of positive feelings such as hope, elation and happiness [31].

#### 2.3.4 World Health Organization Disability Assessment Schedule (WHODAS) 2.0

WHODAS 2.0 was used as a measure to gain insight into the psychosocial functioning of all the participants who were recruited in the study [21]. The questionnaire measures six functional domains viz: cognition, mobility, self-care, getting along with people, life activities and participation in society. WHODAS 2.0 has been reported to have high internal consistency (Cronbach’s coefficient of 0.96 for its 36 items), good test-retest reliability (intra-class correlation coefficient of 0.98), decent validity (correlation coefficients 0.45–0.65), and a reasonable sensitivity (effect size 0.44–1.38) [21]. The standardized scores range from 0 to 100, with higher scores indicating greater levels of disability in normal functioning.

### 2.4 Physiological measures

#### 2.4.1 Waist–hip ratio and BMI

The anthropometric indices were estimated for all the participants. Briefly, the body weight (kg), height (cm), waist-hip ratio was measured and body mass index (kg/m^2^) was calculated.

#### 2.4.2 Central blood pressure and resting heart rate

Central blood pressure (CBP) was measured using USCOM BP+© instrument (USCOM Ltd., Sydney, Australia). USCOM BP+© employs supra systolic oscillometric technology to compute CBP. CBP is non-invasive, reliable and clinically important than peripheral blood pressure in predicting the hemodynamic changes associated with various cardiovascular diseases [32]. As the pulse wave propagates from aorta to the periphery, its characteristics change due to progressive increase in arterial stiffness and decreased arterial diameter. Thus, the systolic peak becomes more prominent and systolic pressure increases as the pressure wave travels from highly elastic central arteries to stiffer peripheral arteries. Briefly, to measure CBP, the participants were allowed to relax for 05 min and using upper arm cuff the brachial arm pressure was measured by inflating the cuff. The automated inflation to supra systolic pressure provided an accurate measure of CBP. Systolic blood pressure (SBP) and diastolic blood pressure (DBP) were recorded during the measurements. Resting heart rate was measured prior to administration of the psychological questionnaires to negate influence of extrinsic stressors like medical examination room environment on the outcome of the questionnaires.

#### 2.4.3 Pulse Wave Velocity (PWV) measurements

Arterial stiffness was assessed by determining brachial-ankle pulse wave velocity (baPWV) using the plethysmographic apparatus (AD instruments) as described previously [33]. In brief, the subjects were laid in supine position after at least 5 min of rest. For the recording of baPWV, pulse waveform of brachial artery and posterior tibial artery was recorded simultaneously with pulse transducers for 5 minutes using Powerlab^TM^ 4/35 hardware and Labchart^TM^ 8 reader software was used to analyse the data (AD Instruments, Sydney, Australia). The system settings for acquisition of data and determination of wave front were: frequency- 1000 Hz; low pass filter- 50 Hz. The time interval between the foot of the wavefront of brachial and ankle waveform was designated as ∆ T_ba_. The distance between the sampling points of baPWV was calculated as: baPWV = (L_a_-L_b_)/∆T_ba_ where, L_b_ = (0.2195 × suprasternal notch to brachium [in cm]–2.0734) and L_a_ = (0.8129 × suprasternal notch to ankle[in cm]+12.328), L_a_ and L_b_ are distances and ∆T_ba_ is pulse transit time.

#### 2.4.4 Measurement of HbA1c, Estimated Glomerular Filtration Rate (eGFR) and Tetrahydrobiopterin (THB)

Blood samples were obtained from all the participants in fasting conditions at baseline and follow-ups. HbA1c was assayed using ion-exchange high-performance liquid chromatography (HPLC).

The eGFR was assessed using the Chronic Kidney Disease Epidemiology Collaboration (CKD-EPI) equation using serum cystatin C and serum creatinine as it provides a much more accurate and precise estimate of GFR [34]. The equation for calculation of eGFR:

$$eGFR=135\times min{\left(Scr/\kappa ,1\right)}^{\alpha }\times max{\left(Scr/\kappa ,1\right)}^{-0.601}\times min{\left(Scys/0.8,1\right)}^{-0.375}\times max{\left(Scys/0.8,1\right)}^{-0.711}\times 0.995^{Age}$$

Where, Scr is serum creatinine, Scys is serum cystatin C, κ is 0.7 for females and 0.9 for males, α is −0.248 for females and−0.207 for males, min indicates the minimum of Scr/κ or 1, and max indicates the maximum of Scr/κ or 1. A factor of 0.969 was used in the equation in case of females.

The estimation of tetrahydrobiopterin was done in the serum samples of the recruited participants spectrophotometrically using ELISA kits according to the manufacturer’s instructions (Cat: MBS283103, My BioSource, USA). Dithiothreitol (5 μl of 1M solution) was added to 250 μl of serum samples to prevent oxidation of thiol group of tetrahydrobiopterin. The absorbance (A_450_) reading of the serially diluted standards was used to plot the standard curve. All the serum samples were run in duplicates and mean of both the readings for a single sample was considered for statistical analysis.

### 2.5 Radioimaging for carotid artery thickness

Thickness of both the right and left internal carotid arteries was assessed using high resolution ultrasound scanner (IU22, Philips) at Department of Radiology, AIIMS, Bhubaneswar. The set-up enabled the measurement of diameter, distension and intima-media thickness (IMT) [35]. The mean IMT of the common carotid artery was measured over a segment of the common carotid artery that was 1 cm long, located approximately 0.5 cm below the carotid-artery bulb and non-plaque portion was chosen. The maximum IMT of the internal carotid artery was defined as the greatest intima–media thickness in either the right (IMT-R) or left (IMT-L) internal carotid artery extending from the bulb to 1 cm above the carotid sinus. Measurements obtained from 10 healthy middle-aged volunteers with no known clinical conditions were taken as reference for normal IMT values.

### 2.6 Statistical analysis

Univariate, bivariate and multivariate analysis was performed for statistical analysis of the study results. Univariate analysis was conducted to analyse profile of study participants at the time of enrolment into the study. Bivariate analysis was conducted at each study time i.e. baseline and follow-up to analyse differences in depression severity (HAMD score), psychological scores, arterial stiffness measures and circulatory biomarkers between treatment responders and non-responders. The differences between treatment responders and non-responders were tested using t-test. Further, for repeated measures Analysis of Variance (ANOVA) was conducted to test the individual effect of treatment response status, time as well as joint effect of treatment response status and time. Mixed method models were fitted to further understand the effect of treatment response on study parameters. These models were adjusted for respondent’s age, sex, education and socio-economic status. Both ANOVA and mixed model results were adjusted for multiple comparison using Bonferroni test. All the analyses were performed using Stata version 15.

## 3. Results

**Fig 2:**
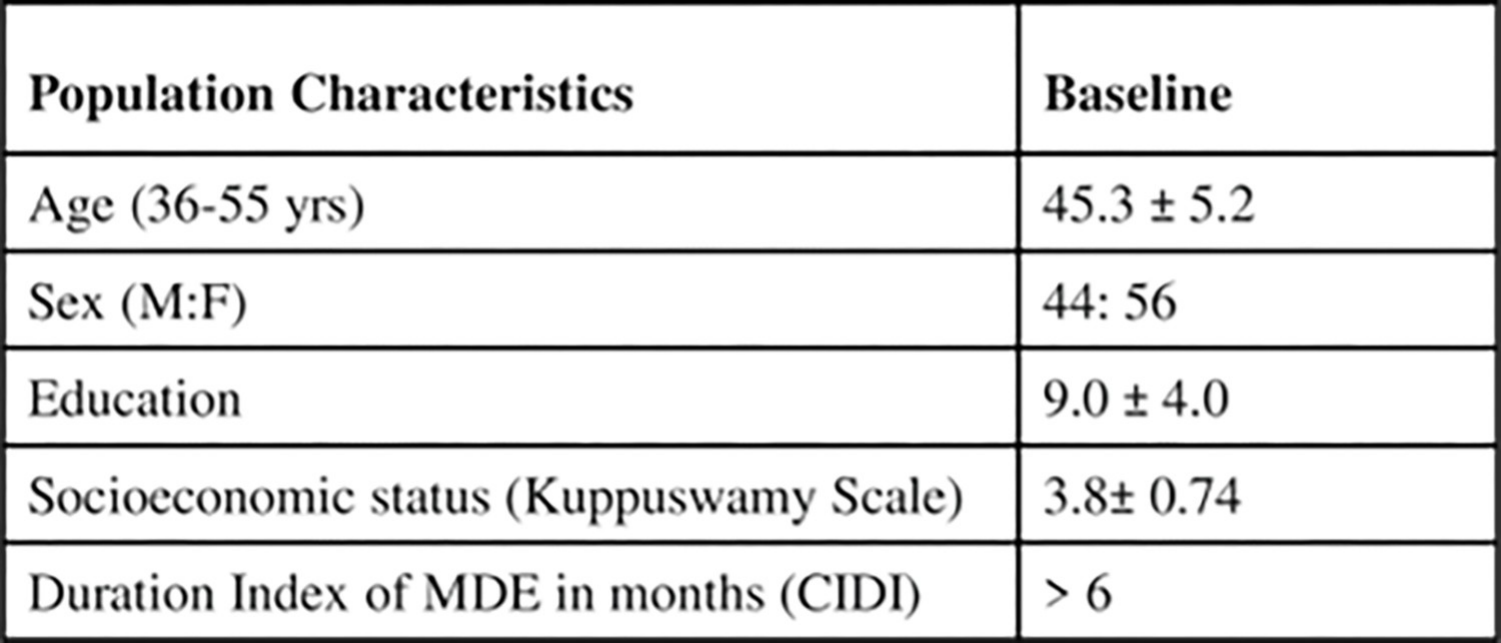
Patient demographic and clinical characteristics (Mean ±SD) of newly diagnosed MDD patients (n = 101) at baseline.

The demographic and clinical characteristics for the MDD patients at baseline are presented in Fig 2. All the volunteers were middle aged, newly diagnosed with MDD, literate and belonging to the lower and upper lower socioeconomic strata.

**Fig 3:**
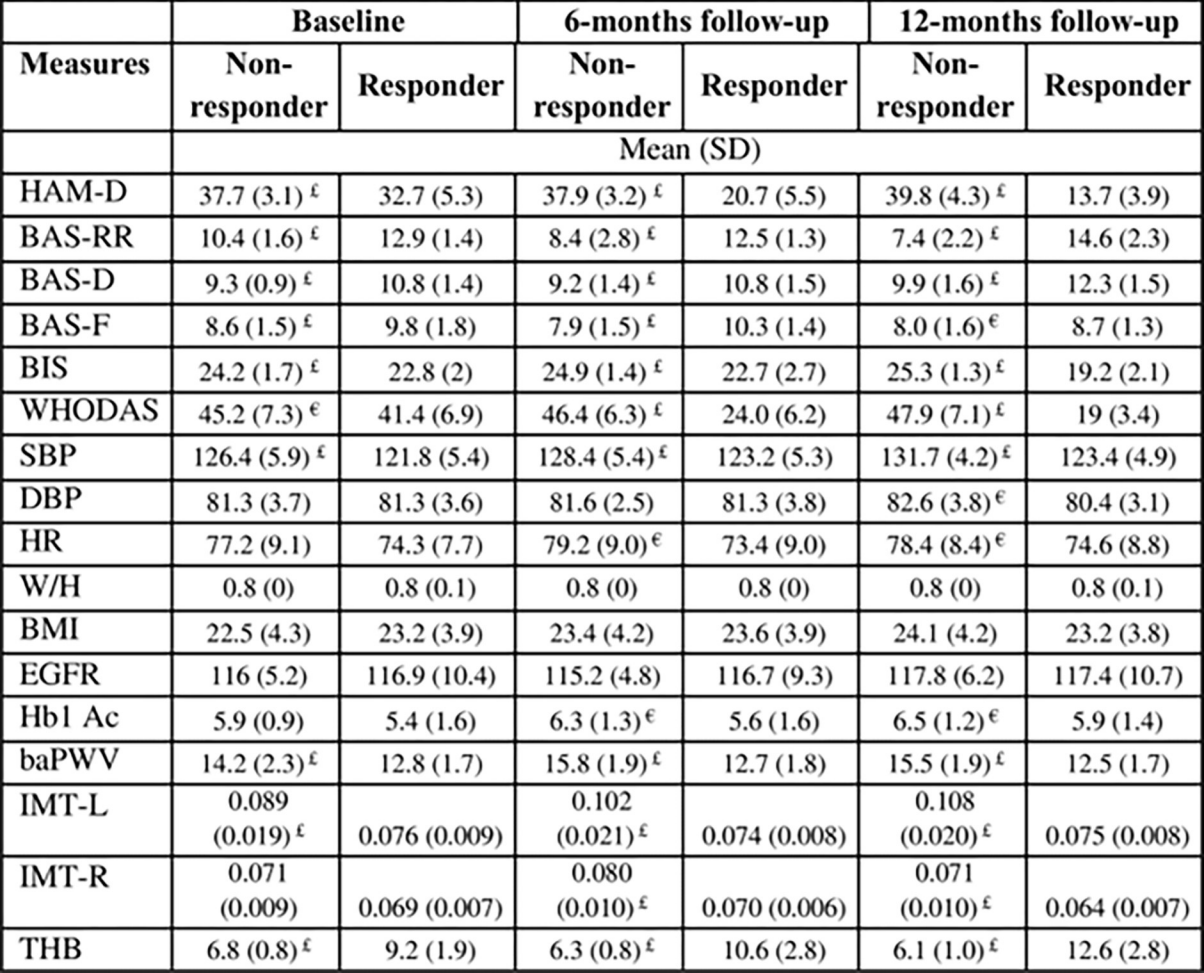
Parametric mean ±SD scores of responders and non-responders to anti-depressant treatment at various study check points. £indicates p < 0.01 and € indicates p < 0.05 between responders and non-responders as determined using t-test.

Comparison of parametric scores between responders and non-responders at baseline and follow ups shows significantly higher HAM-D, BIS, WHODAS, SBP, baPWV and IML values in non-responders, as shown in Fig 3. In comparison to the responders, the non-responders had lower THB, BAS-RR, BAS-D at baseline and follow-ups. The difference in scores between responders and non-responders in WHODAS, BAS-RR, IMT-L and THB widened progressively at 6 months and 12-month follow-ups.

**Fig 4:**
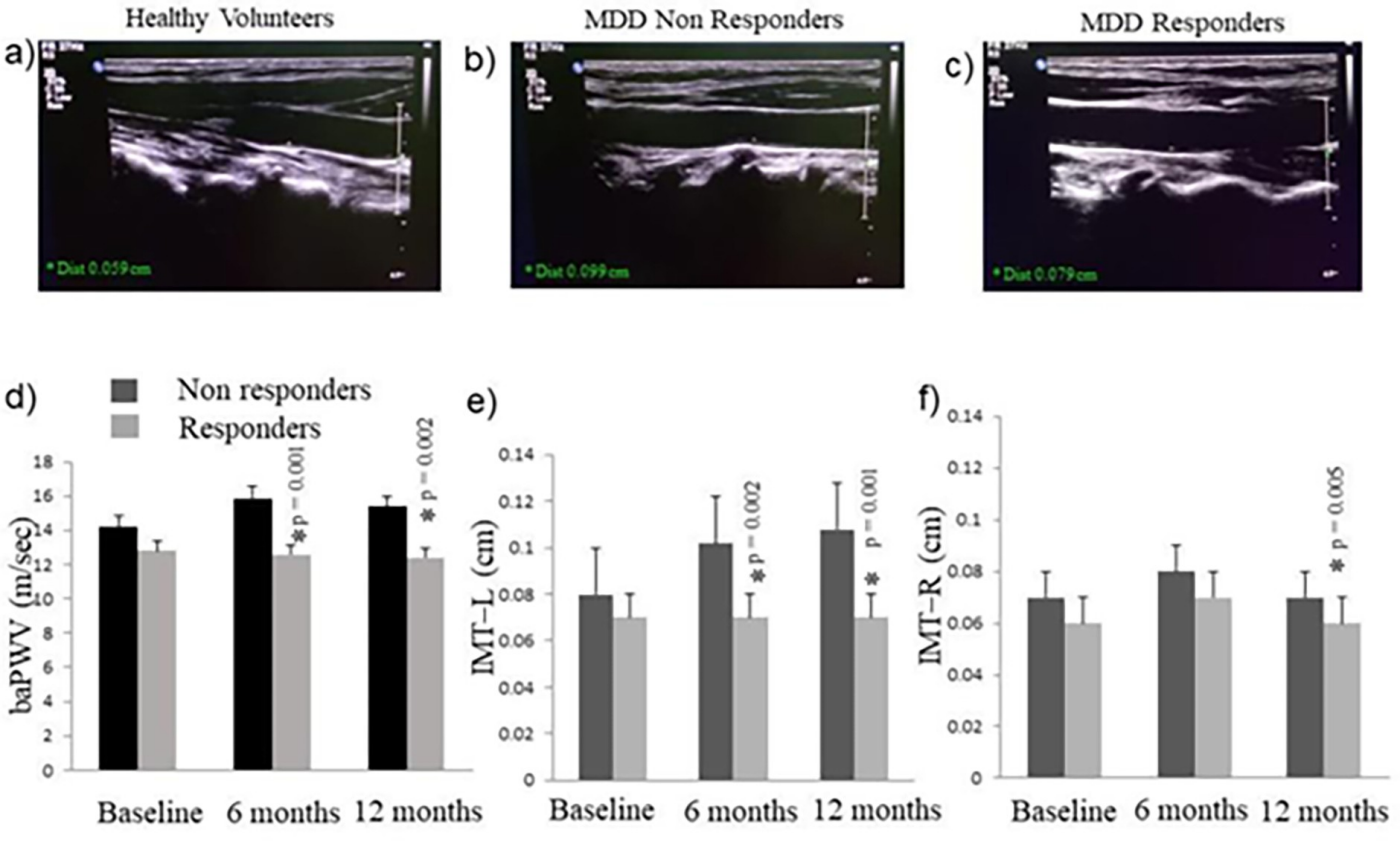
Representative images of intima media thickness in a) healthy volunteers b) Treatment non-responders and c) Treatment responders. Graph denotes Mean ±SD of d) Brachial-ankle pulse wave velocity e) Intima-media thickness of left carotid artery f) Intima-media thickness of right carotid artery.

Measurement of baPWV showed significant difference in mean values between responders and non-responders during baseline and follow-ups as shown in Fig 4. Radio-imaging of left carotid artery, however, showed progressive increase on intima media thickness in treatment non-responders group during follow-ups. Mean IMT-L of non-responders group was higher than the mean intima media thickness of treatment responders during baseline and follow-ups. Non-responder group also had higher mean intima media thickness of right carotid artery at 6 and 12 months follow-up when compared to responders.

**Fig 5:**
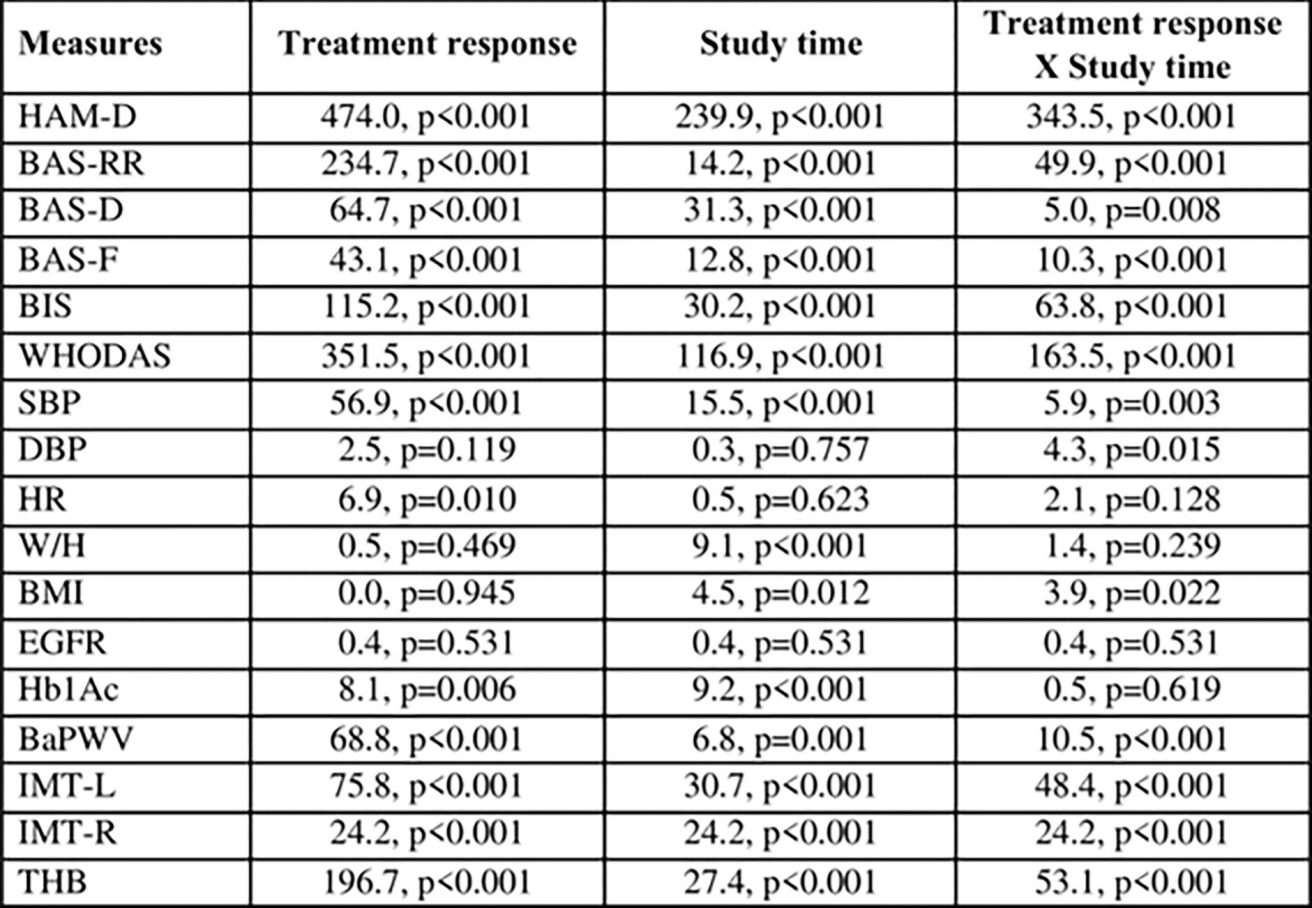
Individual and joint effect of treatment response and study time on various measures. The tests were conducted using repeated measures ANOVA. The p-values were adjusted for multiple comparison using Bonferroni test.

Repeated measures ANOVA suggest that the interaction term of treatment response and study time are statistically associated with almost all study parameters except for HR, W/H, EGFR, and Hb1Ac (Fig 5).

**Fig 6:**
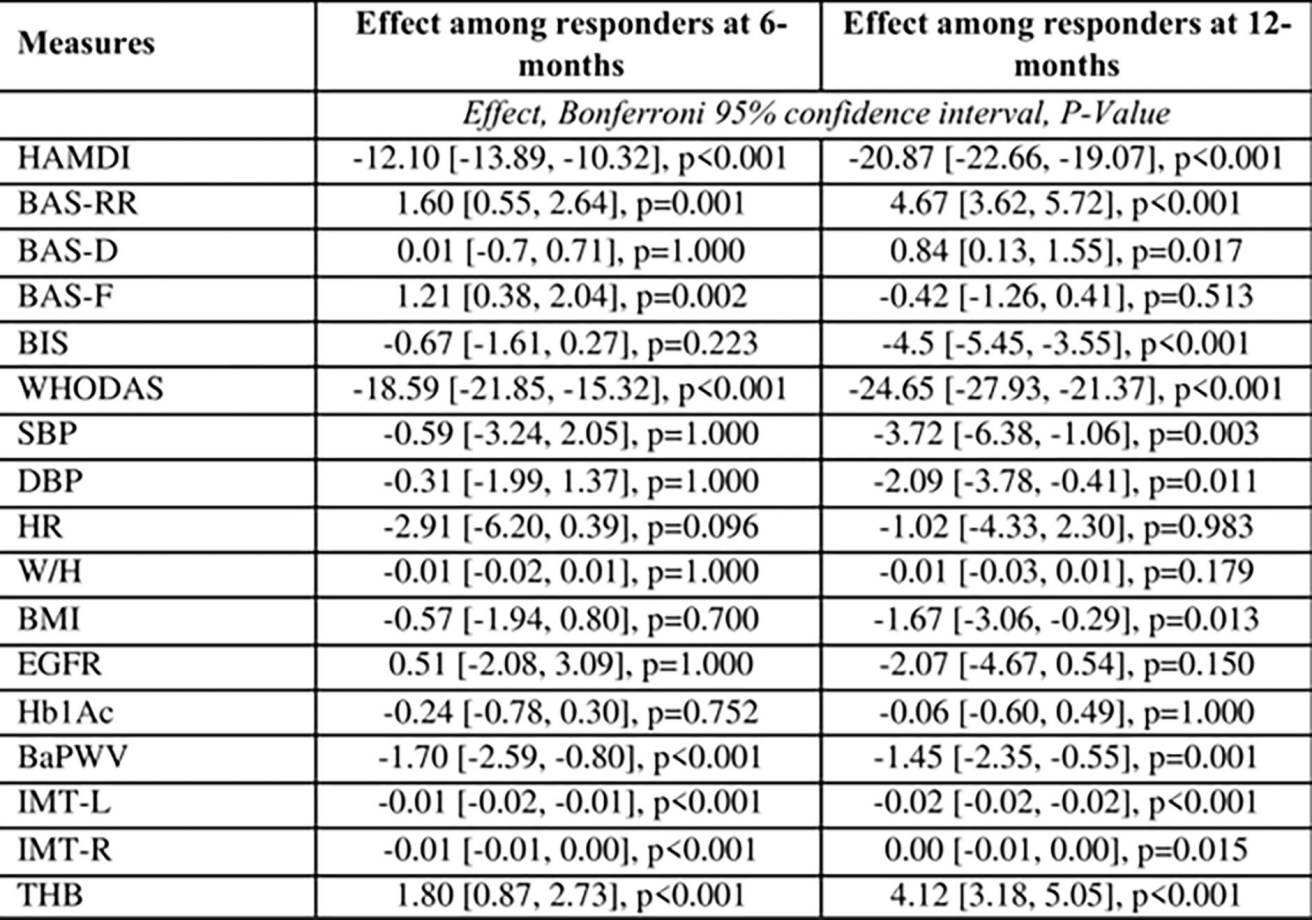
Multivariable mixed effect model assessing the individual and joint effect of treatment response and study time on various measures. The mixed effect models were adjusted for age, sex, education, and socio-economic status. The confidence intervals and p-values were adjusted for multiple comparison using Bonferroni test.

**Fig 7:**
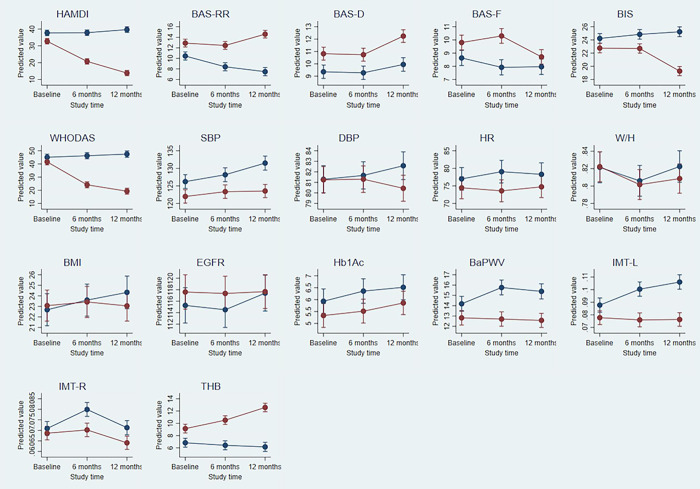
Adjusted predicted values of parameters viz. HAM-D, BAS-RR, BAS-D, BAS-F, BIS, WHODAS, SBP, DBP, HR, W/H, BMI, EGFR, Hb1Ac, BaPWV, IMT-L, IMT-R and THB at different study time.

Similar to the ANOVA, the multivariable mixed model shows that over time, specifically after 12 months, treatment responders has positive effective compared to treatment non-responders (Fig 6). The adjusted predicted value for each parameter by study time is presented in Fig 7. Parameters in which significant changes were noted among treatment responders compared to non-responders at 12 months are HAM-D, BAS-RR, BAS-D, BIS, WHODAS, SBP, DBP, BMI, BaPWV, Hb1Ac, IMR, IML and THB.

**Fig 8:**
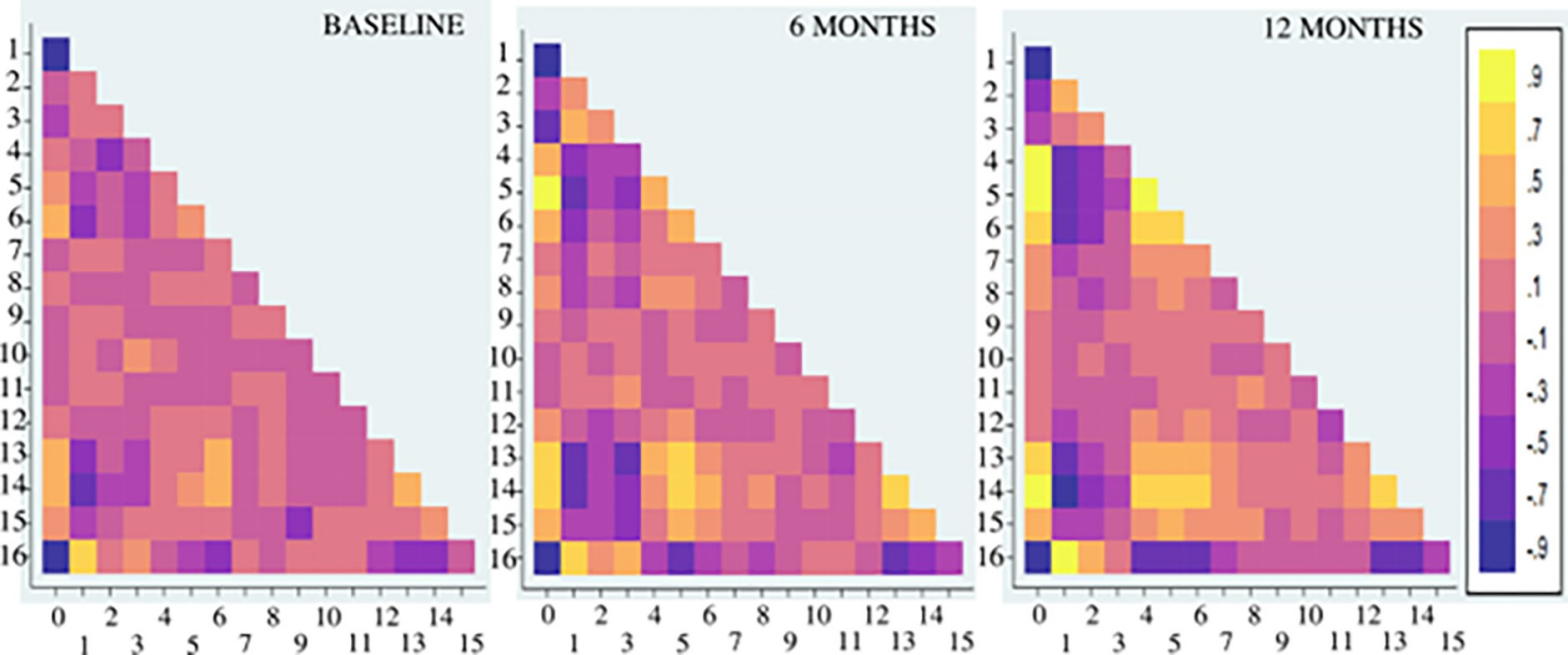
Heat map showing interdependence of study variables on a linear -1 to +1 colour scale with dark purple depicting maximum negative association and bright yellow depicting maximum positive association. Numbers on the x and y axis from 0 to 16 denote HAM-D, BAS-RR, BAS-D, BAS–F, BIS, WHODAS, SBP, DBP, HR, W:H, BMI, eGFR, Hb1Ac, baPWV, IMT-L, IMT-R and THB respectively.

The relationship between study variables at different time points is being depicted in the heat maps in Fig 8. Corelation analysis of study parameters showed high negative correlation of HAM-D scores with BAS-RR at baseline (α = -0.8226), 6 months follow up (α = - 0.8411) and 12 months follow up (α = -0.9325) in the study population as shown in Fig 8. Subjects with higher HAM-D scores had relatively lower BAS-RR scores during baseline and follow ups. A strong negative correlation was also noted between IMT-L and BAS–RR scores in the study population (baseline α = -0.6302, 6 months α = -0.7558 and 12 months α = -0.8137) indicating possible influence of central arterial stiffness on reward responsiveness.

We also observed a positive correlation between IMT-L and HAM-D scores at baseline (α = 0.5292) which increased progressively during follow ups at 6 months (α = 0.7361) and 12 months (α = 0.8319). Similar positive correlation was noted between HAM-D scores and baPWV at baseline (α = 0.4305), 6 months follow up (α = 0.7278) and 12 months follow up (α = 0.7020). A positive correlation was observed between IMT-L and baPWV (baseline α = 0.5289, 6 months α = 0.6935 and 12 months α = 0.6195). SBP on the other hand showed a high correlation with HAM-D scores (α = 0.7544) during 12 months follow up. These findings indicate towards possible association of arterial stiffness with intensity of depression.

The present study showed strong negative correlation of HAM-D scores with tetrahydrobiopterin (THB) at baseline (α = -0.9020), 6 months follow up (α = - 0.8343) and 12 months follow up (α = - 0.8882). IMT-L in the study population was negatively correlated with THB at baseline (α = - 0.5321), 6 months follow up (α = -0.5880) and 12 months follow up (α = -0.7344). THB also showed negative association with baPWV during baseline and follow ups (baseline α = - 0.4787, 6 months α = - 0.6295 and 12 months α = - 0.6982).

## 4. Discussion

Our study provides novel insights on association of early atherosclerosis and central arterial stiffness with motivational systems and severity of depressive traits in middle-aged TRD patients. Though various scores for assessing severity of depression effectively take symptoms into account, the psychological factors like behavioural motivational systems do not form part of the diagnostic criteria. BAS, that regulates the appetitive motives, and BIS, that governs aversive motives, can potentially influence severity of depressive traits and suicidal behaviour. A recent study on suicide attempters suggests that low BAS and high BIS may be associated with depression and suicidal ideation [36]. Similarly, others have identified BAS and BIS dysregulations to be a predisposing factor for depression [37]. However, none of the previous studies have investigated the role of BIS and BAS on treatment resistance in depression. Our findings show significantly lower BAS RR and higher BIS scores in middle-aged patients with TRD when compared to those with remittance. The concomitant increase in WHODAS 2.0 scores with BAS RR values indicated greater influence of reward responsiveness on psycho-social behaviour. Correlation analysis of BIS-BAS parameters with depression severity also showed association of higher HAM-D scores with lower BIS RR (Fig 7B). These findings find support from previous studies showing influence of BIS on depression through intolerance of uncertainty and of BAS through anhedonia [38].

The present study showed differentially higher intima-media thickness of left internal carotid artery in patients with TRD in comparison to MDD responders. Our observation finds support from previous studies showing reduced blood flow to left anterior cingulate, the left dorsolateral prefrontal cortex and the left angular gyrus in subjects with depression when compared to normal subjects [39]. In light of this information, we propose that decreased BAS RR in TRD patients could possibly be due to increased atherosclerosis and altered perfusion in the capillary bed of left internal carotid artery. The strong correlation of intima-media thickness of left internal carotid artery and baPWV with depression severity provides evidence for this hypothesis. Further, our findings on relatively lower intima-media thickness and baPWV in treatment responders strengthen our argument on role of left internal carotid artery atherosclerosis in TRD.

Cerebral perfusion is influenced by endothelial dysfunction, arterial stiffness and other factors affecting vascular health [40]. The diagnostic significance of these factors as contributors to vascular depression, however, remains to be established. A recent study, using applanation tonometry, has shown association of aortic stiffness with MDD and depressive symptoms among middle-aged men [17]. Similarly, increase in baPWV, which provides a measure for central arterial stiffness has been associated with somatic symptoms of depression [41]. Previous studies have shown the association of higher pulse wave velocities with severe depressive symptoms in adolescents [42]. Conversely, others have reported depression to be a significant risk factor for development of atherosclerosis in middle-aged Japanese male subjects [43]. Barring a few studies on young and middle-aged population, the role of arterial stiffness and pulse wave velocity in vascular depression has been largely investigated on geriatric population [44]. The Age, Gene/Environment Susceptibility (AGES)-Reykjavik epidemiological study on geriatric population concluded that greater arterial stiffness is associated with more depressive symptoms. This association of arterial stiffness and depression is partly accounted by white matter hyper-intensity volume and sub-cortical infarcts [45]. Based on these previous reports we suggest a similar role of central arterial stiffness in modulating treatment resistance in middle-aged MDD patients. The association of HAM-D scores with IMT-L and baPWV in the middle-aged study population provides evidence for role of arterial stiffness in severity of depression (Fig 7B).

Central blood pressure, heart rate variability, body mass index, glomerular filtration rate and plasma glucose have been previously reported to be confounding factors for arterial stiffness [43,45,46]. Though these factors are closely inter-related, they can independently influence vascular functions and cerebral perfusion [47]. We, therefore, studied possible association of each of these factors on treatment resistance and severity of depression. Our results show no significant difference in BMI, W/H ratio, eGFR and HbA1c between responders and treatment resistant groups. We, however, observed lower levels of circulatory tetrahydrobiopterin in TRD patients when compared to responders. Tetrahydrobiopterin is a critical cofactor for the production of nitric oxide via endothelial nitric oxide synthase (eNOS) activity, thereby influencing vascular activity. It has recently been emerged both as an important biomarker for reduced microvascular circulation and resultant tissue hypoxia [48]. Interestingly, a progressive decline in circulatory tetrahydrobiopterin was observed in the TRD patients during the present study along with concomitant increase in biopterin. Lower tetrahydrobiopterin concentration was associated with higher depression severity and lower BAS-RR scores in the study population. Similar alterations in total biopterin and active biopterin has been previously reported in patients with affective disorders, schizophrenia and panic disorders [49]. Tetrahydrobiopterin regulates cerebral hemodynamics through production of eNOS that causes vasodilation and improves cerebral perfusion. The remittance from depressive symptoms in patients with higher levels of tetrahydrobiopterin which was observed during the present study could be due to similar eNOS mediated vasodilatory mechanisms.

## 5. Conclusion

Based on our findings, we propose a role of atherosclerosis and central arterial stiffness in defining reward response based motivational behavior and remittance of depression symptoms in middle-aged MDD patients undergoing antidepressant therapy. While association of arterial stiffness with depression is well established in geriatric population, our study provides evidence for involvement of vascular factors even in middle-aged MDD patients. The association of the behavioral approach system with circulatory tetrahydrobiopterin which was observed during the present study provides novel insights into the pathophysiological mechanisms associated with BIS-BAS dysregulation in middle-aged population with TRD. Based on these findings, further investigation on effect of supplementation of tetrahydrobiopterin as adjunct to antidepressant therapy for TRD patients can provide valuable insights towards alternative approaches for clinical management of treatment resistant depression.

Though absence of data on cerebral perfusion is a major limitation of the study, we have relied on previous reports showing differential regional cerebral blood flow in normal and depressed patients^39^. Neuroimaging data in TRD and patients showing remittance could not be obtained because the study was carried out during Covid pandemic period. However, future studies on association of regional cerebral blood flow with treatment resistance and atherosclerosis in middle-aged population can provide further information on its clinical relevance.

## Supporting information

S1 Data (XLS)
